# On the Digestion of Alcoholic Liquids and Their Operation in Nutrition; with Hygienic Applications

**Published:** 1847-02

**Authors:** 


*On the Digestion of Alcoholic Liquids and their operation in Nutrition; with Hygienic Applications.*—M.M. Bouchardat and Sandras, and M. B., alone, have presented some interesting *memoires* on these subjects to the French Academy of Science, from which we will give extracts, as follows:

The first part of the digestive process, which consists in a dissolution, is wanting, with alcoholic drinks, as it also is with fatty substances. Alcoholic fluids undergo no other alteration, in the digestive apparatus, than dilution with the gastric juice and mucus, saliva, and the other liquids which are poured into the digestive organs.

The absorption of alcoholic drinks is effected, as has already been proved by Magendie (*Physiologie*, 3d edition, p. 187,) by the orifices of the veins. It is particularly in the stomach that this absorption takes place: when alcoholic drinks are administered in great excess, or mixed with sugar, this absorption may continue throughout the rest of the intestines.

The chyliferous vessels do not aid in the absorption of alcoholic drinks; after their ingestion the chyle may be very abundantly collected, if these drinks have been given with the fatty aliments: in this case the chyle does not contain any appreciable trace of alcohol.

When alcoholic drinks are introduced into the torrent of the circulation, the alcohol is not eliminated by any of the secretory organs; a small quantity only is evaporated by the lungs, and may be collected with the gases and vapors which are continually exhaled by that organ.

If alcohol is introduced into the circulatory apparatus in too great quantity, the arterial blood becomes of the color of venous blood, and the alcohol may occasion all the accidents of asphyxia.

The alcohol under the influence of the oxygen, which is incessantly introduced into the economy by respiration, may be converted into water and carbonic acid; but, in several of our observations, we have obtained an intermediate product of combustion,—*acetic acid.*

Alcohol and its products rapidly disappear from the economy. When it is introduced simultaneously with grape-sugar or dextrine, its destruction is more rapid than that of these latter bodies.

*The Comparative Action of Alcohol on different Animals.*—When introduced into the torrent of the circulation—it is there that the principal action of oxygen takes place—and the blood globules, being deprived of this vivifying principle, cannot acquire their vermillion color; they are asphyxiated, and if the quantity of alcohol is large the animal dies as though it had been plunged into air deprived of oxygen. The carnivora, as the dog, whose stomach is voluminous compared with the rest of the digestive apparatus, are very sensible to the action of alcohol, and may be killed by a moderate dose; for it is rapidly absorbed without passing beyond the duodenum. The herbivorous gnawers, as rabbits, are also killed by a small quantity of alcohol, for absorption in the stomach is very rapid; alcohol is not to be found in their intestines. The graminivorous birds, such as the domestic fowl, can support large doses; the cavity of their stomach is limited; that organ is furnished with strong muscles; alcohol which is ingested does not remain there: it may be found in the intestines; it is from thence transported to the liver by the venæ portæ, and then gains admittance slowly into the great apparatus of the circulation. Fishes can live at the temperature of 56° centigrade, in water containing the half of one per cent. of alcohol.

*Influence of Alcoholic Drinks on the Secretion of Urine.*—My experiments lead me to admit that, under the influence of a large quantity of alcoholic drinks, the amount of urine rendered in the twenty-four hours is diminished; the same is the case with the absolute quantity of urea; the uric acid, on the contrary, is excreted in larger quantity than the normal proportion.—*Comptes Rendus. Tome.—W. Lancet.*

